# Knowledge, attitudes and practices of men in a South African rural community in relation to exclusive breastfeeding

**DOI:** 10.4102/safp.v64i1.5366

**Published:** 2022-02-11

**Authors:** Oscar M. Mabele, Matthew O.A. Benedict, Wilhelm J. Steinberg, Elizabeth Reji, Cornel van Rooyen, Anthonio O. Adefuye

**Affiliations:** 1 Department of Family Medicine, Faculty of Health Sciences, University of the Free State, Bloemfontein, South Africa; 2 Department of Biostatistics, Faculty of Health Sciences, University of the Free State, Bloemfontein, South Africa; 3 Division of Health Sciences Education, Faculty of Health Sciences, University of the Free State, Bloemfontein, South Africa

**Keywords:** exclusive breastfeeding, knowledge, attitudes, practice, adult males, South Africa

## Abstract

**Background:**

Several lifelong maternal, child and societal health benefits have been associated with exclusive breastfeeding (EBF). However, despite all the potential advantages, EBF rates have been consistently low in developing countries, including South Africa. It has been suggested that the knowledge, attitudes and practices of male partners in relation to EBF are amongst the important factors that contribute to the success of EBF practices. Hence, the aim of this study was to determine the knowledge, attitudes and practices of men in Botshabelo, Free State province, South Africa, regarding EBF.

**Methods:**

This study was designed as a cross-sectional analytical study that utilised a structured questionnaire administered to 200 adult men attending the outpatient department of a district hospital, in the Free State province, South Africa.

**Results:**

The majority (*n* = 83; 41.5%) of participants had poor knowledge of EBF but reported positive attitudes (*n* = 153, 76.5%) and good practices (*n* = 151, 75.5%) towards EBF, respectively. Age, levels of education, employment status, marital status and whether the participant accompanied his partner to the antenatal clinic were associated with adequate knowledge, positive attitudes and good practices in relation to EBF (*p* < 0.05).

**Conclusion:**

The study revealed a suboptimal level of knowledge on EBF in men in Botshabelo. Most men had positive attitudes and reported good practices in relation to EBF. Our findings highlight the need for targeted community-based intervention programmes directed to educating and promoting positive social and cultural change in relation to EBF amongst men in Botshabelo.

## Background

Exclusive breastfeeding (EBF) could be the primary source of food for infants and could support optimal growth and development for the first six months of life. Several lifelong maternal, child and societal health benefits have been associated with EBF.^1^ Recent data suggest that EBF can substantially mitigate the risk of both short- and long-term health defects, such as stunted growth and impaired cognitive ability.^2^ Similarly, optimal breastfeeding has been reported to prevent around 12% of deaths in the under 5 years age group every year, amounting to around 800 000 lives being saved in low- and middle-income countries.^3^ However, despite all the potential advantages, the practice of EBF is consistently low in sub-Saharan Africa.^4^ Factors such as poor understanding and cultural perceptions of EBF, as well as conflicts between traditional beliefs and EBF, have been identified as barriers to the sustainability of EBF on the continent.^5,6^

In South Africa, there is a paucity of recent and reliable data on breastfeeding rates in the country. A 2016 South Africa demographic and health survey (SADHS) report indicates that there has been a four-fold increase in the number of women who exclusively breastfeed their babies – from 8% in 2003 to 32% in 2016.^7^ However, despite this progress, South Africa still has one of the lowest breastfeeding rates on the continent.^8^ Available national data suggest that most mothers initiate breastfeeding after birth but very few practise EBF during the first six months post-partum, despite numerous government policies, guidelines, regulations and legislative instruments aimed at protecting, promoting and supporting EBF.^9,10^

Data that show that EBF is practised significantly more by married women suggest that spousal support could influence vital decisions, such as whether a woman practises EBF.^11,12^ Similarly, fathers or male partners are identified as an important source of support in the implementation of breastfeeding.^13^ Moreover, it has been reported that a baby has a higher chance of being exclusively breastfed if the father has more knowledge regarding EBF.^14^ This suggests that knowledge and attitude of male partners and fathers in relation to EBF are amongst the important factors that contribute to the success of EBF practices. The aim of this study was to assess the knowledge, attitudes and practices of men in Botshabelo community, Free State province, South Africa regarding EBF. We envisioned that findings from this study would inform guidelines to enhance men’s support for EBF practice in this community.

## Methods

This study was designed as a cross-sectional analytical study.

### Population size and study setting

The target population consisted of adult males (≥ 18 years) who attended the outpatient department of a district hospital, Free State, South Africa. The hospital is a public hospital in a community of about 181 712 people.^15^ The hospital serves as the referral centre for 13 primary healthcare clinics. An average of 70 patients are seen daily at the outpatient department, 21% (*n* = 15) of whom are adult males.

### Sampling

A convenience sampling method was adopted for this study, and data were collected twice a week. At the time of this study, the estimated number of men ≥ 18 years old living in Botshabelo was *N* = 26 000 (sample frame).^15^ The estimated minimum sample size was calculated as *n* = 268 (at 90% confidence level [CL] and margin of error [MOE]: 0.05).^16^ The study population consisted of consenting men who were at least 18 years old, irrespective of whether they were married or had children. A colour-coded sticker system was used to ensure that no eligible male participant could participate in the study more than once (i.e. a colour-coded sticker was placed on a participant’s folder once the participant had completed the questionnaire).

### Questionnaire

The development of the structured questionnaire that was used in this study was informed by findings from prior studies,^17,18^ after a thorough literature review and content analysis. Search terms, such as EBF, adult males and exclusive breast-feeding and knowledge of and attitudes of men towards EBF, were used to search for relevant articles during the literature review process. The databases used to access articles were Google Scholar, MEDLINE, PubMed, CINAHL, SABINET, Science Direct and Directory of Open Access Journals. Concepts were identified to formulate closed-ended questions, which were answered using an adapted Likert-scale ranking. The questionnaire was self-administered, made available in three languages commonly spoken in the local community (i.e. English, Sesotho and Setswana) and distributed manually. The self-administered questionnaire comprised five distinct sections. Section A of the questionnaire obtained data on participants’ eligibility to participate in the study. The demographic and socio-economic details of the participants were captured in Section B of the questionnaire. Questions in Section C assessed participants’ knowledge of aspects of EBF, whilst data on participants’ attitudes and practices regarding EBF were captured in Sections D and E, using a five-point Likert scale, that is, strongly agree, agree, not sure, strongly disagree and disagree.

### Data collection

Prospective participants were briefed about the study by one of the researchers (O.M.M.). Participants were informed that they could choose to participate in the study, or not, and that failure to participate would not compromise their treatment at the hospital. A detailed explanation about the study (also contained in an information leaflet) was given to participants who gave verbal consent. Participants were also informed that implied consent was being given by agreeing to complete the questionnaire. One of the researchers (O.M.M.) and/or a research assistant assisted participants who were incapable of completing the questionnaire on their own. Participants were instructed to drop the completed questionnaire in a sealed box situated in the front desk at the outpatient department. O.M.M. emptied the box daily and all completed questionnaires were locked in a safe. Data from the questionnaires were inputted on a Microsoft Excel sheet on a computer with password protection. The questionnaire had no trace of identification, to ensure that data were collected anonymously. The study was conducted over a period of two months (August 2020 – September 2020).

### Pilot study

We performed a pilot study before the official start of data collection to test the suitability of the study design and methods, the chosen data collection method and the overall structure of the questionnaire. The pilot study consisted of 10 eligible adult men who were selected using convenient sampling. No changes to the structured question resulted from the pilot study. The estimated time needed to complete the questionnaire was 30 min.

### Data analysis

Questionnaire items were scored to determine the percentages of correct or expected and incorrect or unexpected responses. The scoring range of the knowledge questions was 10 (maximum) to 0 (minimum). A score of ≥ 70% (7/10) was considered ‘adequate knowledge’, scores between 50% (5/10) and 60% (6/10) were considered ‘average knowledge’ and a score of ≤ 40% (4/10) was regarded as ‘poor knowledge’. Attitude towards EBF was assessed by obtaining participant responses to 16 items using a five-point Likert scale. For the purpose of this study, ‘strongly agree’ and ‘agree’ responses were summed as ‘agree’, whilst the ‘strongly disagree’ and ‘disagree’ responses were summed as ‘disagree’. The expected responses to the attitude items 1–8 were ‘disagree’, whilst the expected responses for items 9–16 were ‘agree’. There were 10 items in the practice section, and participants’ responses were obtained using a five-point Likert scale. The five-point Likert scale was collapsed to three, that is, ‘Agree’, ‘Disagree’ and ‘Not sure’. Participants who scored ≥ 50% (≥ 5/10) were regarded as having ‘good’ EBF practices, and those who scored < 50% (< 5/10) were considered as having ‘poor’ EBF practices. The expected response for practice items 3 and 4 in the practice section was ‘disagree’, whilst the expected response for the other items in this section was ‘agree’.

The data were analysed using Statistical Analysis System (SAS) version 9. Descriptive statistics (e.g. medians) was used for continuous variables, whilst frequencies and percentages were computed for categorical data. The associations between demographic data and knowledge, attitudes and practice scores were assessed using chi-square or Fisher’s exact tests. A *p*-value of < 0.05 was taken to be significant.

### Validity

The validity of the structured questionnaire was examined by comparing the questionnaire elements with those of previous and similar studies, as well as by conducting the pilot study. A departmental evaluation committee, consisting of consultant family physicians and a biostatistician, subjected the questionnaire to review and approval.^19^

### Ethical considerations

The Health Sciences Research Ethics Committee of the University of the Free State (UFS-HSD2020/0324/2508) granted approval for the study. Further approval was obtained from the Head of the Free State Department of Health.

## Results

### Sociodemographic profile of participants

Two hundred adult males participated, out of an approximate 240 participants who presented during the study period, giving a response rate of 83%. One third (*n* = 64; 32%) of the study participants were in the age category 31–40 years, with a median age of 37 years (minimum = 18 years; maximum = 65 years). In total, 56 (28%) participants completed high school, whilst 54 participants (27%) had some secondary-level education and 51 participants (25.5%) had undergone tertiary-level education. The majority (*n* = 188; 94%) were of the Christian faith. Sixty-eight (34%) of the participants were married, 45 (22.5%) of them were single and 42 (21%) were living with their partners. The majority (*n* = 158, 79%) were of Southern Sotho ethnicity. Regarding employment, 106 participants (53%) had jobs and the majority were skilled/highly skilled. About 50% of them received an annual income ≥ R10 000.00. Of the 200 participants, 148 (74%) had children – the majority had more than one child – and 84 (56.8%) of these fathers had attended antenatal care with the mothers of their children (Table 1).

**TABLE 1:** Sociodemographic characteristics of the participants.

Characteristics	*n*	%
**Age of respondents (years)**		
18–30	57	28.5
31–40	64	32.0
41–50	32	16.0
51–60	35	17.5
> 60	12	6.0
**Highest level of education**		
No formal education	8	4.0
Primary level education (Grades 1–7)	14	7.0
Primary school (Grade 7 completed)	17	8.5
Secondary-level education (Grades 8–11)	54	27.0
Grade 12 (completed high school)	56	28.0
Tertiary	51	25.5
**Religion**		
Atheist	8	4.0
Christian	188	94.0
Hindu	1	0.5
Muslim	1	0.5
Other	2	1.0
**Marital status**		
Married	68	34.0
Divorced	19	9.5
Separated	16	8.0
Cohabiting/living together (Civil union)	42	21.0
Single	45	22.5
Widowed	10	5.0
**Ethnicity**		
Khoisan	1	0.5
Ndebele	1	0.5
Southern Sotho	158	79.0
Tswana	23	11.5
Xhosa	12	6.0
Zulu	3	1.5
Other	2	1.0
**Employment status**		
Employed	106	53.0
Unemployed	94	47.0
**Skill level (*n* = 106)**		
Unskilled	1	0.9
Semi-skilled	19	17.9
Skilled	65	61.3
Highly skilled	21	19.8
**Annual income (*n* = 106)**		
None	89	44.5
< R5 000.00	5	2.5
R5000.00 – R10 000.00	5	2.5
R10 001.00 – R15 000.00	23	11.5
R15 001.00 – R20 000.00	26	13.0
R20 001.00 – R30 000.00	18	9.0
> R30 000.00	34	17.0
**Fatherhood**		
Children	148	74.0
No children	52	26.0
**Number of children (*n* = 148)**		
1 child	35	23.6
2 children	54	36.5
> 2 children	59	39.9
**Accompanied mother to antenatal visit (*n* = 148)**		
Yes	84	56.8
No	64	43.2

### Assessment of knowledge of exclusive breastfeeding

Table 2 describes the responses of the participants to the EBF knowledge questions. Knowledge was assessed by asking questions about aspects of EBF, such as duration of EBF and the effect of EBF on a baby’s immunity. It was found that 65 (32.5%) participants had adequate knowledge, whilst 52 (26%) and 83 (41.5%) participants had average and poor knowledge of EBF, respectively (Tables 2 and 5).

**TABLE 2:** Responses to exclusive breastfeeding knowledge items.

Knowledge question	*N*	%
**For how long should a child drink only breast milk?**		
3 months	19	9.5
6 months†	86	43.0
12 months	61	30.5
I don’t know	34	17.0
**How soon after birth can a mother start bre astfeeding?**		
Immediately after birth†	96	48.0
24 h after birth	55	27.5
Only when the baby starts crying	25	12.5
I don’t know	24	12.0
**When can you start giving additional foods and/or fluids to a breastfed baby?**		
3 months	27	13.5
4 months	3	1.5
5 months	4	2.0
6 months†	98	49.0
12 months	42	21.0
I don’t know	26	13.0
**What foods and/or fluids do the Department of Health recommend you give a baby younger than six months?**		
Plain water only	2	1.0
Breast milk and water	26	13.0
Breast milk only†	86	43.0
Formula milk only	1	0.5
Formula and breast milk	16	8.0
Porridge and breast milk	44	22.0
Formula and porridge	14	7.0
Unsure	11	5.5
**In your opinion, is breast milk alone enough for a baby during the first six months of life?**		
Yes†	105	52.5
No	54	27.0
Unsure	41	20.5
**How does exclusive breastfeeding change a baby’s immunity?**		
Increase immunity†	175	87.5
Decrease immunity	3	1.5
No difference	4	2.0
Unsure	18	9.0
**Breast milk is more easily digested by a baby than formula milk**		
True†	152	76.0
False	2	1.0
Unsure	46	23.0
**How does exclusive breastfeeding impact or affect the mother’s weight?**		
Increase her weight	21	10.5
Decrease her weight	34	17.0
No impact or effect on her weight†	95	47.5
Unsure	50	25.0
**What are the ideal food and/or fluids for a baby older than six months of age?**		
Plain water only	3	1.5
Breast milk and water	12	6.0
Breast milk only	32	16.0
Formula milk only	2	1.0
Formula and breast milk	17	8.5
Porridge and breast milk†	83	41.5
Formula and porridge	7	3.5
Cow milk	1	0.5
Unsure	43	21.5
**What foods and/or fluids can you give a 3-month-old baby who cannot stop crying?**		
Breast milk and water	20	10.0
Breast milk only†	77	38.5
Formula milk only	1	0.5
Formula and breast milk	9	4.5
Porridge and breast milk	18	9.0
Formula and porridge	2	1.0
Unsure	73	36.5
**Total knowledge score (out of 10)**		
0	7	3.5
1	13	6.5
2	20	10.0
3	24	12.0
4	19	9.5
5	25	12.5
6	27	13.5
7	24	12.0
8	21	10.5
9	19	9.5
10	1	0.5

† , The correct answer.

### Assessment of attitudes towards exclusive breastfeeding

Participants who scored ≥ 50% (≥ 8/16) of the 16 attitude items were regarded as having positive attitudes towards EBF, whilst those who scored < 50% (< 8/16) were regarded as having negative attitudes towards EBF. Our findings reveal that the majority (*n* = 153; 76.5%) of the participants had positive attitudes towards EBF (i.e. had scores of ≥ 8/16), whilst 47 (23.5%) had negative attitudes (i.e. scores of < 8/16; Tables 3 and 5).

**TABLE 3:** Participants’ attitudes towards exclusive breastfeeding.

Attitude items	Agree		Disagree		Not sure	
	*n*	%	*n*	%	*n*	%
Breast milk only is not ideal food for babies during their first year of life.	41	20.5	94	47.0	65	32.5
Breastfeeding is only beneficial to a child younger than six months of age.	46	23.0	92	46.0	62	31.0
A baby of three months needs water and other fluids to prevent thirst.	37	18.5	91	45.5	72	36.0
Parents should start giving additional foods to a baby before the baby is six months old.	30	15.0	105	52.5	65	32.5
Mothers should give more breast milk to a boy child as compared to a girl child.	15	7.5	145	72.5	40	20.0
Formula-fed babies are more likely to gain weight more quickly than breastfed babies.	17	8.5	108	54.0	75	37.5
You can give a baby younger than six months both breast milk and formula milk if they cannot stop crying.	37	18.5	92	46.0	71	35.5
Formula feeding is more convenient than breastfeeding.	10	5.0	143	71.5	47	23.5
Breastfeeding is more convenient than formula feeding.	160	80.0	10	5.0	30	15.0
Breastfeeding mothers should be helped with household chores.	152	76.0	12	6.0	36	18.0
Breastfed babies are healthier than formula-fed babies are.	156	78.0	11	5.5	33	16.5
It is very important to provide emotional support and encouragement to the breastfeeding mother.	157	78.5	12	6.0	31	15.5
Fathers should be directly involved with breastfeeding activity.	142	71.0	13	6.5	45	22.5
It is important to give breast milk to a newborn baby after birth.	98	70.0	13	9.3	29	20.7
Fathers should use their paternity leave days to assist with the caring and nursing of the baby.	153	76.5	11	5.5	36	18.0
Breastfeeding mothers should be granted maternity leave during the first six months of a baby’s life.	155	77.5	13	6.5	32	16.0

### Assessment of practices relating to exclusive breastfeeding

In total, 151 (75.5%) participants reported good practices in relation to EBF, whilst 49 (24.5%) participants reported having poor practices (Tables 4 and 5).

**TABLE 4:** Practices related to exclusive breastfeeding.

Practice items	Agree		Disagree		Not sure	
	*n*	%	*n*	%	*n*	%
I should be actively involved during the breastfeeding period.	149	74.5	22	11.0	29	14.5
I should encourage my partner to breastfeed.	171	85.5	11	5.5	18	9.0
I feel neglected when my partner is breastfeeding the baby.	35	17.5	85	42.5	80	40.0
The decision to breastfeed should be taken by the mother and not the father.	30	15.0	107	53.5	63	31.5
My partner goes through emotional issues during the breastfeeding period.	90	45.0	20	10.0	90	45.0
I should encourage my partner to practise exclusive breastfeeding.	148	74.0	21	10.5	31	15.5
I should allow my partner to breastfeed, whilst I provide the income to the house.	143	71.5	17	8.5	40	20.0
My presence during breastfeeding time of my child improves the family bond.	138	69.0	11	5.5	51	25.5
I should provide emotional support to the mother of my child/children during the breastfeeding period.	155	77.5	11	5.5	34	17.0
I allow my partner to express her own breast milk in a bottle so that I can feed the baby when she is resting and give her some time off.	119	59.5	29	14.5	52	26.0

### Summary of findings of knowledge, attitudes and practice assessment

Table 5 provides a summary of outcomes of the assessment of knowledge, attitudes and practice in relation to EBF.

**TABLE 5:** Outcome of knowledge, attitudes and practices assessment.

Item	*n*	%
**Knowledge**		
Adequate	65	32.5
Average	52	26.0
Poor	83	41.5
**Attitudes**		
Positive towards EBF	153	76.5
Negative towards EBF	47	23.5
**Practices**		
Good	151	75.5
Poor	49	24.5

EBF, exclusive breastfeeding.

### Knowledge, attitudes and practices of exclusive breastfeeding according to sociodemographic strata

After having obtained participants’ responses on aspects of knowledge, attitudes and practices regarding EBF, we investigated the association between participants’ sociodemographic characteristics and their knowledge, attitudes and practices regarding EBF through bivariate analysis using chi-square or Fisher’s exact tests. The findings, as presented in Table 6, show that adult men in the age group 18–30 years were more knowledgeable of EBF than the other age groups (*p* = 0.001). In addition, adult males who had completed Grade 12 and tertiary education had significantly more knowledge than participants with lower levels of education (*p* = 0.001). We found a significant association between marital status and practice of EBF, as participants who were married had better practices of EBF (*p* = 0.027). Furthermore, the results show that adult men who had attended antenatal visits with their partners were significantly more knowledgeable and had a more positive attitude towards EBF (*p* = 0.000 and 0.001, respectively).

**TABLE 6:** Association between sociodemographic characteristics and knowledge, attitudes and practice of exclusive breastfeeding by the participants.

Background characteristic	Adequate knowledge (%)	Average knowledge (%)	Poor knowledge (%)	*p*	Positive attitudes (%)	Negative attitudes (%)	*p*	Good practices (%)	Poor practices (%)	*p*
**Age category (years)**				0.001*			0.210			0.223
18–30	49.2	25.0	14.5		26.8	34.0		25.8	36.7	
31–40	35.4	36.5	27.7		34.6	25.5		34.4	26.5	
41–50	6.2	23.1	19.3		18.3	8.5		18.5	8.2	
51–60	6.2	15.4	26.5		15.0	23.4		15.9	20.4	
> 60	3.1	0.0	12.0		5.2	8.5		5.3	8.3	
**Level of education**				0.001*			0.193			0.335
No formal education	1.5	0.0	8.4		3.3	6.4		4.0	4.1	
Some primary	4.6	3.8	10.8		5.2	12.8		7.9	4.1	
Primary completed	1.5	5.8	15.7		7.8	10.6		7.3	12.2	
Some secondary	18.5	30.8	31.3		25.5	31.9		23.8	36.7	
Grade 12 completed	36.9	34.6	16.9		30.1	21.3		30.5	20.4	
Tertiary	36.9	25.0	16.9		28.1	17.0		26.5	22.4	
**Marital status**				0.027*			0.568			0.025*
Married	27.7	38.5	36.1		36.6	25.5		37.1	24.5	
Divorced	4.6	9.6	13.3		9.8	8.5		10.6	6.1	
Separated	10.8	7.7	6.0		7.8	8.5		8.3	6.1	
Cohabiting	33.8	17.3	13.3		20.9	21.3		22.5	16.3	
Single	20.0	26.9	21.7		19.6	31.9		17.9	36.7	
Widowed	3.1	0.0	9.6		5.2	4.3		3.3	10.2	
**Employed?**				0.001*			0.137			0.503
Yes	72.3	59.6	33.7		54.9	46.8		55	46.9	
No	27.7	40.4	66.3		45.1	53.2		45.1	53.1	
**Do you have a child or children?**				0.013*			0.003*			0.049*
Yes	61.5	84.6	77.1		79.1	57.4		77.5	63.3	
No	38.5	15.4	22.9		20.9	42.6		2.5	36.7	
**Number of children**				0.001*			0.011*			0.189
1	23.1	26.9	8.4		20.9	8.5		17.9	18.4	
2	30.8	36.5	19.3		30.1	19.1		30.5	18.4	
> 2	7.7	23.1	51.8		29.4	31.9		30.5	28.6	
**Attended antenatal clinic**				0.001*			0.001*			0.150
Yes	47.7	55.8	30.1		49.0	21.3		45.0	34.7	
No	13.8	30.8	49.4		31.4	38.3		33.8	30.6	

* , *p* = ≤ 0.05.

## Discussion

It has been reported that the knowledge of a male partner (or father) about breastfeeding can directly influence mother’s attitude towards EBF.^20^ Whilst fathers having a better knowledge of breastfeeding have been found to lead to increased support for mothers to practise EBF as recommended,^21^ fathers possessing little knowledge of breastfeeding have been reported to be associated with negative practices, such as formula feeding by mothers.^14^ The findings were that the majority of participants possessed little knowledge of EBF, which confirms similar findings by Kavela (2007),^22^ and suggest that these men may negatively influence their partners’ EBF practices. It has been suggested that a simple educational intervention can increase men’s knowledge of breastfeeding and, thereby, increase rates of breastfeeding in the first six months after a baby’s birth.^23^ It is, therefore, plausible that targeted education of these men will enhance their knowledge of EBF and thereby their partners’ EBF practices.

We found that married men, and those who cohabited with their partners reported having adequate knowledge of EBF, compared to the other relationship statuses (*p* = 0.027); however, only 27.7% of those who reported to be married had adequate knowledge. The lower-than-expected number of married men who reported adequate knowledge could be related to the majority (61.8%) of these men being aged older than 41 years and reporting a lower level of education (45.6%). This is in accordance with findings by Jimoh (2004), who reports that younger, married men are more knowledgeable about EBF and will encourage their wives or live-in partners to breastfeed exclusively.^24^ The present study also found that participants with children had adequate knowledge, positive attitudes and good practices towards EBF (*p* = 0.013, *p* = 0.003 and *p* = 0.049, respectively). This is in accordance with findings of studies, which report that most fathers have good knowledge of and positive attitudes towards EBF.^17,25^

The positive association between men’s age (being younger), a high level of education and knowledge of EBF has been documented in the literature.^26,27^ Similarly, we found that younger men (aged 18–40 years) who had completed Grade 12 and had tertiary education had significantly more knowledge of EBF than older men (≥ 41 years) and men with lower levels of education (*p* = 0.001). This finding is corroborated by findings of earlier studies, by Aniebue et al. (2015) and Banu et al. (2012).^27,28^ In addition, we found that younger men (aged 18–40 years) had more positive attitudes about and better practices regarding EBF than older men. Although this finding was not statistically significant, it suggests that younger men are more knowledgeable about EBF than older men and would more likely to support their partners to practise EBF.^24^ Our findings that show no statistically significant difference between men with higher levels of education and those with lower levels of education regarding positive attitudes and good practices to EBF suggest that educational status is not the only determinant of men’s attitudes to EBF and that a high level of education may not encourage positive attitudes or good practices. Other factors, such as misconceptions and cultural beliefs about breastfeeding, have been reported to influence men’s support for EBF.^22^ The belief that breastfeeding is bad for a woman’s breasts, that breastfeeding makes breasts look ugly, that breastfeeding separates the baby from the father, that colostrum is bad milk, that breastfeeding is only a woman’s issue and that ‘real’ men do not talk about or support breastfeeding are some of the misconceptions and cultural beliefs that prevent men from promoting, supporting and protecting EBF.^17,18,27^ It is, therefore, very likely that even some well-educated men in Botshabelo honour these beliefs about breastfeeding, which indicates the need for more targeted, community-based interventions to promote positive social and cultural change that will promote positive attitudes about EBF amongst men in Botshabelo.

Furthermore, the data show that married men and those who cohabited with their partners had better practices and more positive attitudes in relation to EBF than men who reported other relationship statuses and living arrangements. This suggests that married men or men who cohabited with their partners were most likely to get involved in and support EBF, in keeping with findings by Silva et al. (2012).^29^

It has been reported that male partner participation in antenatal care is important and contributes to more positive maternal and neonatal birth outcomes.^30^ The present study found that men who attended antenatal care with their partners had adequate knowledge of and a positive attitude towards EBF – better than men who did not attend antenatal care with their partners (*p* = 0.001 and *p* = 0.022, respectively). This finding is in agreement with reports by researchers that suggest that including men in antenatal care visits can enhance their knowledge of and support for EBF.^30,31^ This could mean that men who attended antenatal care with their partners received education about and teaching on the importance of EBF, which could have changed their perceptions of EBF.

In conclusion, this study found a suboptimal level of knowledge on EBF in adult men in Botshabelo. The majority of adult men had positive attitudes and reported good practices in relation to EBF. The study findings expose the need for more targeted, community-based interventions and programmes directed to raise awareness of the benefits of EBF in men in Botshabelo. Health practitioners in Botshabelo should consider involving male partners in both antenatal and postnatal programmes on EBF. We envisage that inculcating the right knowledge of and attitudes towards EBF in these men will enhance the practice of EBF in the local setting.

### Strengths and limitations

As far as we know, this is the first study executed in Botshabelo that investigated the knowledge, attitudes and practice regarding EBF of men. Several limitations of this study should be noted. Firstly, only health-seeking adult men were included in the study. It is possible that there are other adult men in the Botshabelo community with varying levels of knowledge and different attitudes and practices regarding EBF, thus, limiting the generalisability of this study. Secondly, because of time constraints and the advent of the COVID-19 pandemic, only 200 participants were surveyed; the involvement of a greater number of participants could, perhaps, have made the findings more representative of the community. The fact that non-random sampling was used could also be considered as a limitation of this study.
